# Do Austrian “INTE﻿GRI (integrated care) projects” comply with international definitions and concepts?

**DOI:** 10.1007/s10354-017-0555-5

**Published:** 2017-03-20

**Authors:** Isabel Geiger, Claudia Wild

**Affiliations:** 1 MCI/Management Center Innsbruck, Universitätsstraße 15, 6020 Innsbruck, Austria; 2 Ludwig Boltzmann Institute of Health Technology Assessment (LBI-HTA), Garnisongasse 7/20, 1090 Vienna, Austria; 3 University of Liverpool, Liverpool, UK

**Keywords:** Integrated care, Key elements, INTE﻿GRI award, Integrierte Versorgung, Hauptkriterien, INTE﻿GRI-Award

## Abstract

One of the biggest challenges for European healthcare systems is the fragmentation of care. To overcome this challenge, integrated care (IC) approaches have been recently implemented. To further improve this method, current and past projects must be monitored and evaluated. However, since the definition of IC is very indistinct and varies significantly in literature, key elements have to be defined. The study design selected was a mixed-methods study that includes two approaches: a systematic literature review and qualitative content analysis of the data provided by the Ludwig Boltzmann Institute. Nine key elements of IC projects were identified in the literature review and subsequently compared with the main features coded from previous INTEGRI applications. The results showed that 41 of the applications presented seven or more criteria in their official submission form. The conclusion of the results can be drawn as a justification and validation of the INTEGRI criteria. Although the results are positive on the whole, three recommendations on possible improvements are given.

## Zusammenfassung

Die Fragmentierung des Gesundheitssystems innerhalb eines Landes stellt eine der größten Herausforderungen europäischer Gesundheitswesen dar. Eine von vielen Ländern anerkannte Methode zur Bekämpfung dieser Herausforderung ist das Konzept der integrierten Versorgung. Um diese Methode weiterzuentwickeln und zu verbessern, ist es vonnöten, Hauptkriterien für Projekte in diesem Bereich zu bestimmen. Als Studiendesign wurde hierfür eine „mixed-methods study“ gewählt, welche 2 verschiedene Ansätze beinhaltet: einen systematischen Literaturüberblick und eine qualitative Inhaltsanalyse der Daten zu INTEGRI-Projekten, welche vom Ludwig-Boltzmann-Institut zur Verfügung gestellt wurden. Das Ergebnis des Literaturüberblicks ergab 9 Hauptkriterien für integrierte Versorgungsprojekte, welche anschließend mit den wichtigsten Charakteristika der INTEGRI-Projekte verglichen wurden. Es wurden mindestens 4 übereinstimmende Elemente gefunden, wobei 41 von 52 Projekten 7 oder mehr Elemente beinhalteten. Die Zusammenfassung dieser Resultate kann als Validierung und Rechtfertigung der gefragten Kriterien zur Einreichung des INTEGRI-Awards angesehen werden. Trotz der überwiegend positiven Resultate wurden 3 mögliche Verbesserungsvorschläge ausgearbeitet.

## Introduction

Very recently The King’s Fund published a paper on the effects (challenges and impact) of new or extended roles in delivering integrated care (IC) [1]. Once again this article highlights the importance of IC as a possibility to overcome the challenges of European healthcare systems, which are currently struggling with the fragmentation of care, disjointed pathways for patients resulting in a higher rate of adverse hospitalisation, diagnostic workup, medical errors and inferior outcomes, in addition to the changing medical needs of people [2].

Austria, as one example of a highly fragmented healthcare system, has made efforts in its “Federal Targeting-Agreement for Health 2013–2016” to stimulate IC projects and activities. To give attention to those efforts the INTEGRI award, as one example, was launched in 2011 with its main purpose of honouring professionals and organisations that contribute with innovative ideas, tender projects tackling the challenges of a constantly changing health environment and assist in improving the Austrian healthcare system through integrated care approaches [3, 4]. When INTEGRI was awarded for the first time (2012), a total number of 36 submissions supported the preliminary assumption that IC was already highly valued in Austria and many professionals devoted their work to it. By 2014, an additional 17 applications were submitted, resulting in a total of 53 different projects.

To further improve IC approaches in the future, current and past projects must be monitored and evaluated. However, since the definition of IC is very indistinct and varies significantly in literature, the evaluation of such projects appears to be very challenging. To enable a valid, replicable and transparent evaluation process, key elements have to be defined.

The purpose of this research was to conduct accompanying research to the INTEGRI award. The following paper will give a systematic literature review on published concepts, guidelines, principles and recommendations established by the WHO (World Health Organisation) and the European Union to identify key elements of IC projects. In addition, this paper will apply the criteria used for assessing the INTEGRI applications to contribute to the advancement (and objectification) of the Austrian award along international standards.

## Methodology

The research is based on a mixed-methods study, including two approaches: a systematic literature review and qualitative content analysis of the data (INTEGRI award submissions).

For the *literature review*, a comprehensive literature search in three electronic bibliographic databases and search engines was performed on the 22 February 2016 using PubMed, Embase and ScienceDirect. The following combinations of terms were used for detecting appropriate literature: “integrated care” AND “evaluation”, “integrated care” AND “principles”, “delivery of health care, integrated” and “integrated care” AND “policy”. Additionally, the *International Journal of Integrated Care* (IJIC) and the *Journal of Integrated Care* (JICA) were handscreened systematically. Also, hand search for grey literature, especially for those published by the WHO Regional Office for Europe, the European Commission and the European Parliament was carried out.

Predefined selection criteria for inclusion were (1) published between 2011 and 2016, (2) availability in English or German and (3) description and explanation of key elements, features or criteria for evaluating IC projects. Exclusion criteria were all articles which (1) mainly focused on reporting clinical interventions and outcomes, (2) pilot studies or (3) studies conducted in middle- or low-income countries.

About 3000 studies were identified and after removing duplicates, 1052 were screened (abstracts only). Subsequently, 120 full texts were acquired for further detailed investigation. Finally, after excluding another 101 articles and including studies obtained by hand search (*n* = 7), 26 articles met the inclusion criteria and were therefore used to identify key elements [5].

The *assessment of the quality *of the 26 included publications was conducted in a mixed form: For assessing the quality of literature reviews, the AMSTAR checklist [6] was used. Due to the lack of validated quality assessment tools for other study types, ranks between ‘high’, ‘medium’ and ‘low’ were assigned using a modified version of Harden’s quality assessment [7], categorising the publications on three quality levels. Additionally, all studies included were assigned to four categories, beginning with the most relevant studies (1) literature reviews and mixed-study methods including reviews, (2) policy papers, (3) framework analysis, rationales and reports and (4) journal articles, commentaries and declarations. To enable comparability between different categories, the AMSTAR scores were also divided into ‘low’ (score 0–3), ‘medium’ (4–7) and ‘high’ sections.

To examine both the literature review and the analysis of the data (INTEGRI award submissions) in a systematic way, a *qualitative content analysis* approach based on Philipp Mayring was used [8]. As a first step, the category definition and the level of abstraction were decided. Once central rules of category formation were defined, all 26 publications were analysed carefully and essential phrases were highlighted. Each important phrase was listed (and clustered if necessary) and respective categories were formulated. To check whether the categories fit to the research question and also to make sure that all central aspects were noted, a revision of all articles was performed. In an iterative process of categorisation and revision, certain categories were combined and others were split into separate topics. Following the revision, a final coding of important phrases was carried out, starting from the beginning of the articles for enhancing “intra-coder agreement” [8].

The analysis of the INTEGRI award submissions was carried out in the same manner, with identical rules. However, after coding important phrases, listing and revising them, no new categories were formulated. Instead, the project characteristics were matched deductively with the key elements in the final part of the paper. For clarity and comprehensibility, project phrases describing similar aspects were merged.

## Results

### Literature review

Publication characteristics and quality: Eleven publications were identified and classified as category one (*n* = 11: literature reviews, 42%), representing the largest group [9–19], of which the scores of the AMSTAR checklist ranged from two to eight points (low to medium quality). Three articles were classified as category two (*n* = 3: policy papers, 12%) ranging from ‘low’ [20] to ‘medium’ [21, 22] quality. Nine publications were listed under category three (*n* = 9: framework analysis, rationales and reports, 34%) equally distributed between ‘low’ [23–25], ‘medium’ [26–28] and ‘high’ quality [29–31]. Category four (*n* = 3: journal articles, commentaries and declarations, 12%) appeared to have the lowest quality with one ‘medium’ quality article [32] and two publications which both classified as ‘low’ quality [33, 34]. The majority of the selected articles (*n* = 16, 60%) were funded by public organisations or health institutions such as the WHO and Ministries of Health. Additionally, about 80% of the publications were conducted in Europe (*n* = 21), specifically in the Netherlands (*n* = 5), Belgium (*n* = 4) and Denmark (*n* = 3).

Key Elements of Integrated Care: Table 1 provides an overview of each key characteristic identified in the literature analysis, its frequency and the relevant studies. For a better understanding of the elements, they are briefly described. Importantly, since some of the categories have been mentioned more often than others, they are additionally ranked.

**Table 1:** Key elements of integrated care as described in recent publications (2011–2016; *n* = 26) and applied to INTEGRI submission (2012, 2014; *n* = 53)

Key elements	Frequency in literature (*n* = 26) (References)	Ranking (1–9)	Key elements applied to INTEGRI submissions (*n* = 53) (Projects)	Ranking (1–9)
People empowerment and focus on patient	*n* = 20 [9, 11, 13–17, 19, 21–24, 26–31, 33, 34]	1	*n* = 51 [1–16, 18–23, 25–53]	3
Change management and governance	*n* = 20 [9, 13, 15–23, 25, 27–34]	2	*n* = 36 [1, 3, 4, 7, 8, 11, 14, 15, 18, 21, 23–26, 28, 29, 31, 33–36, 38–40, 42–53]	8
Common care strategies	*n* = 19 [9, 12, 14–23, 25–30, 34]	3	*n* = 48 [1–3, 5–18, 20–32, 34–36, 38–48, 50–53]	5
Workforce development	*n* = 17 [9, 12–14, 14–19, 21, 22, 24, 25, 27, 28, 30, 34]	4	*n* = 49 [2–8, 10–17, 19–23, 25–53]	4
Enabling and supportive environment	*n* = 17 [9, 11, 14, 15, 17–23, 27–30, 32, 34]	5	*n* = 38 [1, 4, 5, 7, 9, 10, 12, 13, 15–20, 22, 24, 26–29, 31–34, 37–48, 50, 53]	6
Uniform information and communication technology	*n* = 16 [9, 11, 12, 15–23, 25, 27, 30, 31]	6	*n* = 23 [1, 6, 8, 11, 13, 16, 18, 20, 25–27, 31, 33–35, 40, 42, 45, 46, 48–50, 52]	9
Innovative financing	*n* = 14 [9, 11, 12, 14, 17, 21–23, 25, 26, 30–33]	7	*n* = 52 [1–48, 50–53]	1
Clear goals and persistent evaluation	*n* = 14 [9, 10, 15–18, 20, 21, 23, 25–27, 30, 31]	7	*n* = 52 [1–48, 50–53]	1
Continuity of care	*n* = 10 [9, 12, 14, 16, 20, 27, 29–31, 34]	9	*n* = 37 [1, 3–5, 7, 9–11, 14, 16, 17, 22, 27–45, 47, 48, 50–53]	7

*People empowerment and focus on patients: *One out of two most frequently (*n* = 20) mentioned elements is people or patient empowerment defined by providing support in self-management, emphasising patient education, individual skill development and supporting people to enable deliberate decisions [17]. Furthermore, healthcare providers should see them not only as their patients, but rather as partners in attaining the common goal of better health [29]. To additionally increase people’s quality of life, the use of individualised care (also referred to as personalised care planning [13]) with a central focus on patient’s health needs and preventive measures was also emphasised.

*Change management and governance* (*n* = 20) is described as explicit, but flexible management, as well as integrated governance. Another feature referred to was the implementation of specific change management strategies to decrease or handle people’s potential resistances to change [21]. Also, as Nicholson et al. [17] pointed out, a strong commitment to strengthening clinical leadership and improving accountability by defining responsibilities and coordinating services can be seen as additional attributes of IC projects.

*Common care strategies* (*n* = 19), as a key feature of IC that not only focuses on the outcome, but also concentrates on the care delivery process itself [26]. Mitchell et al. [12] stressed the creation and implementation of care pathways; other authors point to evidence-based guidelines/protocols [27] to align policies as substantial instruments [15].

*Workforce development *(*n* = 17) refers to professional integration and the development of multidisciplinary teams. Mitchell et al. [12] highlight the importance of the right mixture of interdisciplinary professionals and clearly defined roles and responsibilities for facilitating an intra- and extramural communication and cooperation. Furthermore, a part of professional development is ongoing education and training, either in the area of IC, familiarising the different professionals with common strategies and values of joint working, or in exchanging knowledge of different healthcare providers [17].

*Enabling and supportive environment *(*n* = 17): Having an environment with supportive legislations and policies enabling the implementation of IC models was repeatedly mentioned. Specifically, incentivising the delivery of IC, either by incentives for performance or by generating commitment, is pointed out by Lyngso et al. [15]. Other possible approaches include engaging stakeholders by implementing round-tables [20], supporting a paradigm shift and integrating patients in their communities by, e. g., involving their families [28].

*Uniform information and communication technology *(*n* = 16): The need for a universally applicable clinical information system is mentioned as of relevance. Lyngso et al. [15] expressed the importance of a centralised patient record system for enhanced and efficient information flow. This, however, requires the willingness to share information and a high level of trust of all stakeholders involved [12]. Moreover, to achieve an end-to-end information exchange, a standardised, specifically dedicated software was claimed to be useful [23].

*Innovative financing *(*n* = 14): Like in any other sector, IC projects also need to have adequate, viable financing methods to be sustainable [32]. Community-based finance models were described as an example for the cost-efficient use of resources [26]. Martinez-González et al. [16] stress the potential option for IC projects to pool funds across several levels of care.

*Clear goals and persistent evaluation *(*n* = 14): For evaluating the effects of IC, clear goals are necessary. van Houdt et al. [18] state explicitly the importance of identifying clear goals and defined target groups. Additionally, measurement tools for recording quality improvement and/or performance and health outcomes should be implemented [31].

*Continuity of care *(*n* = 10): Importantly, continuity of care has to be seen from a patient’s point of view and refers to his/her perception of a coherent and comprehensive care delivery process [31]. Providing equitable access, preferably by a single point of entry and smooth transitions between different care-providers, are also noted frequently [27]. Moreover, having consistency in health professionals is seen by the WHO [31] as a decisive factor for enhancing user satisfaction and providing patients with a positive experience which, in turn, should facilitate better health outcomes.

### INTEGRI award submissions and application of key elements

Project characteristics: 53 INTEGRI award submissions were analysed: 36 different projects descriptions submitted in 2012 (68% of all) and a further 17 (32%) from 2014. The length of the applications was between 5 and 27 pages. The descriptions included general information, epidemiology, goals, methods, integration, patient centeredness, transferability, cost–benefit relation, quality management, communication and marketing concept, concept for evaluation, evaluation results, room for improvements and attachments for supplementary information. Most applications were carried out by public institutions (*n* = 23) like regional hospitals or social care homes, which represented 43% of all submissions. A number of the 49 projects (92.5%) are currently ongoing or have already been carried out and evaluated. Only 7.5% (or four candidate submissions) were still in the phase of developing ideas.

Key elements in the description of the INTEGRI submission (projects): Table 1 provides a comparison between each key characteristic identified in the literature analysis and applied to the submitted projects and their frequency in the respective 53 submissions, described here in their order of giving weight to the key elements.

*Clear goals and persistent evaluation *(*n* = 52): The project intentions for evaluating outcome or success are varying in quantity between one to six concrete goals, and are omnifarious, including, e. g. the usage of synergies or improvements in efficiency in diagnostics and therapy. In all, 35 submissions are intending to improve the quality of care, comprising aspects like enhanced survival rates or specialised treatments. For evaluation purpose, e. g. feedback is acquired through patient- and relative-questionnaires or through cooperation with universities or other external analysts.

*Innovative financing *(*n* = 52): Efficient re-allocation of acquired savings implies transparency in costing and billing. Such savings can be earned—as claimed in the submissions—with fewer needed transports, shortened hospital stays and/or fewer (re-)admissions by decreasing the number of emergencies, complications and recurrences of diseases (revolving door effect). Additionally, the relief of other sectors, the avoidance or delay of early exit of a working life, as well as the reduction of sick leaves contribute to the need for innovative ways of resource management, since optimised financial management contributes to the sustainable usage of resources by avoiding redundancies in services. Therefore, the projects suggest good documentation of costs and avoided costs.

*People empowerment and focus on patient *(*n* = 51): Most projects explicitly declare to be patient-centred or patient-oriented, including the provision of individualised services based on patients’ needs, and offering direct patient–doctor dialogues. Additionally, enhanced quality of life through, e. g. the improvement of patients’ satisfaction, improved safety in treatment and less suffering of patients, as well as higher survival rates, is expected. Several projects highlighted the importance of regaining patients’ autonomy and maintaining their self-responsibility. The provision of detailed information and issuing clear instructions to enable self-management is implemented. In addition, a few applications claimed to emphasise patient empowerment through enhanced education and training.

*Workforce development *(*n* = 49): Most projects include components of communication, professional integration and multidisciplinary teamwork. The enhancement of intra- and extramural cooperation by fostering the communication between the different sectors is stressed. The improvement of multiprofessional teamwork includes the integration of social services, care nurses and general practitioners (GP) and the offering of multidisciplinary support. Advanced educational programmes comprising new learning techniques and methods, knowledge exchange in multiprofessional workshops and meetings, problem-based learning as well as cross-sector training to improve the level of knowledge in all services are offered in the projects.

*Common care strategies *(*n* = 48): Most submissions not only incorporate standardised assessments of the patients, including predefined parameters, indicators and measures, but also standardise the processes of service delivery by implementing treatment pathways or care plans and by developing clear guidance on different levels of care. The compliance with evidence-based guidelines or with best practices models is frequently stressed.

*Enabling and supportive environment *(*n* = 38): The cooperation between all stakeholders and their involvement in the process of developing common goals is mentioned as a major enabler and organised in most projects. Furthermore, the integration of relatives, e. g. by offering educational programmes or psychological support to ease their burden, as well as other surrounding factors like the destigmatisation of mental diseases and the sensitisation of the environment, as well as the inclusion of the communities and the social environment by offering public relations (PR) activities to avoid future conflicts, are emphasised. Financial incentives to enhance the compliance of GPs are mentioned for facilitating cooperation.

*Continuity of care *(*n* = 37): Particularly the provision of equitable access, as well as a coherent and comprehensive care delivery process with consistent personnel by granting gapless documentation and an end-to-end cooperation, are planned and organised in most submissions. Securing the access to care by removing barriers like waiting times and thereby easing the admission process, or by offering additional treatment and prompt diagnosis are activities within the projects.

*Change management and governance *(*n* = 36): Clearly defined and agreed upon responsibilities, definitions of work-flow and tasks as well as explicit (disease- and discharge-) management or integrated governance (case management) and interface management between different sectors are stated in the applications. The service coordination throughout different levels of care and specific change management activities in implementation is also stressed by several projects.

*Uniform information and communication technology *(*n* = 23): Universally applicable information and communication technologies (ICT) with uniform medical records are mentioned in about half of the submission. Solutions are, e. g. a central database, telemedicine, common electronic documentation or specific software connecting different sectors.

### Comparison of INTEGRI award submissions with literature key elements

After the analysis of the 53 INTEGRI submissions, grouped characteristics (features) were created whilst comparing the coded phrases with the literature (see Table 1). The results illustrate that a minimum of four out of nine elements were met by each INTEGRI project. Furthermore, 41 of all INTEGRI submissions presented seven or more key criteria in their application. One-third of the submissions (*n* = 18, 34%) included eight key elements. Five of the elements were mentioned by 90% and more. In specific, those most frequently mentioned were “innovative financing” as well as “common goals and persistent evaluation”; each was only missing in one project. In more detail, the majority mentioned the feature of “specific goals” also in the context of cost savings, re-allocation of resources and efficiency and therefore showed an overlap with “innovative financing”. “Patient empowerment and focus on patient” was mentioned in 51 submissions, most often described as patient-centred. “Enabling and supportive environment”, “continuity of care” and “change management and governance” were incorporated by 60–80% of all projects.

## Discussion

### Summary and discussion of findings

IC is a very complex and multifaceted area which tries to connect different stakeholders throughout all sectors of health. Although efforts on enhancing the taxonomy have already been made, e. g. by Valentijn et al. [9], there is still no uniform definition available. Consequently, identifying key elements out of an indefinable subject is challenging and requires certain delimitation. For that reason, the literature review was restricted in time, language and type of study. Nevertheless, the elements identified are still relatively widespread and give an ample scope of interpretation. This limiting factor might have negatively impacted the results presented in this paper.

In this respect, it has to be further mentioned that all analysed documents, including the articles from literature and the project submission forms, were carefully read, reread and revised to enhance the “intracoder agreement”, also referred to as intratester reliability. But, to comply with the guidelines based on Mayring [8], it would have been valuable if a second analyst had been involved to improve the interrater reliability. Another possible approach in this regard would have been including a computer-based programme to analyse all documents. However, due to time restrictions and the scope of this paper, neither a computer-based system nor a second examiner was included in this research.

The results of this paper contribute to the understanding of IC by contrasting the theoretical concepts, approaches, guidelines, principles and recommendations with real-life examples. Those projects include the implementation and organisation of IC for chronic diseases and/or a successful integration of social care with the healthcare sector. More specifically, the results show that almost all key characteristics were mentioned by about 70% and more of the projects (*n* ≥ 36). This can most probably be attributed to the submission form, which already requires several key elements itself (patient-centeredness, clear goals and an evaluation concept, including cost–benefit assessment and quality management, costing and billing solutions).

Although having a template aided many applicants in submitting a comprehensive report, the analysis also showed that it was not only beneficial. Uniform information and communication technology (ICT), for instance, was found in less than 50% of the project descriptions. The fact that ICT is not separately mentioned as a specific area in the official submission form could be suspected as one of the reasons. Other explanations for the low quantity of projects describing ICT, however, could also be the novelty of this approach. Also, if no information technology (IT) system has ever been established, implementing a new one would be an expensive investment. Furthermore, as Mitchell et al. [12] highlighted, having a uniform IT platform needs a high level of trust and willingness of all stakeholders to share information. This constitutes a big issue in Austria exemplified during the implementation phase of the electronic medical health record (ELGA), where medical doctors, especially GPs, launched a campaign asking patients to refrain from ELGA for data protection reasons [35].

Another element that was not separately required on the application form, but could be found within other sections was “change management and governance”, which was described rather implicitly by less than 70% applicants with defining responsibilities, etc. Nonetheless, not only due to the fact that change management and governance was most frequently mentioned in literature, but also because of the high number of aspects it includes, it might be focused on more extensively in future award applications (as much as in setting-up the projects).

The economic impact of IC projects was not included, neither within the key elements nor in the project analysis, although terms like cost efficiency and cost savings as aspects of innovative financing were repeatedly found. The underlying reasons for that consist of the complexity of an impact evaluation and the lack of appropriate data. However, obtaining profound evidence/data on the estimated amount in savings and cost efficiency would be desirable for future analysis. It would especially be of essence because the effects of IC approaches and their economic impact are still unclear. Having data on real-life projects in this area could support researchers like Nolte and Pitchforth [2], who already made significant efforts in gathering information on utilisation, cost effectiveness and costs/expenditures.

### Limitations

The literature review aimed at identifying key elements of IC projects through a systematic literature research. Although the electronic database search was supplemented with a hand search for grey literature, publication bias cannot be ruled out. As noted earlier, the lack of international taxonomy of IC constitutes another limitation, which in turn may reduce the validity of the search terms used. Moreover, although scientific articles are usually published in English, some relevant literature could have been missed because of language restrictions. All articles included highlighted certain aspects of IC projects, which have later been coded to identify the key elements. However, only a small number of studies had their primary focus on the evaluation of IC. The assessment of the INTEGRI submissions was based on a qualitative content analysis approach. However, biases due to potential, unwitting personal selectivity cannot be completely ruled out. Furthermore, the analysis performed was based on theoretical and qualitative elements only. There was no quantitative evaluation due to the lack of appropriate data and time restrictions.

## Conclusion

The main contribution of this paper is the determination of the criteria used for assessing the INTEGRI applications. The underlying aim was to validate these criteria and to contribute to the advancement and objectification of the Austrian award along international concepts and definitions. To fulfil this purpose, key elements from literature were compared with the information provided by the 2012 and 2014 INTEGRI participants. Special focus in analysing the projects was put on the official submission forms, primarily to see if the information which had to be provided corresponds to the aspects identified in the literature. As the results show, all applications include at least four out of nine key elements. Most elements, in turn, were mentioned by 70% of the projects and more. Additionally, having a mean of seven incorporated elements can also be seen as an achievement. Therefore, the conclusion of the results can be drawn as a justification and validation of the INTEGRI criteria.

It is not only an acknowledgement for the INTEGRI award itself, but also for Austrian policy makers in healthcare. Their previous efforts to implement IC as a response to the current challenges of the health system have shown to be successful. Although the results are positive on the whole, three recommendations on possible improvements are given:

One aspect found to be lagging behind others and which should be considered for future developments of the official submission form was uniform information and communication technologies. It would be advisable to include this in the template as a separate area, like patient centeredness.

Another suggestion concerning the adaptation of the submission form is to enclose additional parts of the element change management and governance. A new version could also include questions about defining responsibilities within the project and how change management will be/was implemented.

It is also recommended that if future INTEGRI awarding wants to honour not only innovative approaches in the field but also contribute to the advancement of the research in this area, the economic impact of IC projects should be outlined as well. A possible suggestion for modifying the cost–benefit requirements would be to add specific questions on innovative ways of financing.

In general, further research on the definition of IC should be carried out to facilitate international comparison. Importantly, search terms used should then also include “stepped care” and other synonyms of IC. Moreover, and also regarding the recommendation above, additional research on the real economic impact of IC and propositions on how to measure it accurately is recommended.
